# An Integrated Circumferential Proportion and Defect Length Index for Predicting Esophageal Stricture After ESD in Large Mucosal Defects ≥6 cm

**DOI:** 10.3390/jcm15145445

**Published:** 2026-07-11

**Authors:** Li Tang, Yuxiang Chen, Qing Lu, Zhu Wang, Jinlin Yang, Yuzhi Liu, Kai Deng

**Affiliations:** Department of Gastroenterology and Hepatology, West China Hospital of Sichuan University, Chengdu 610041, China

**Keywords:** esophageal stricture, endoscopic submucosal dissection, mucosal defect circumferential proportion, length-to-residual mucosal proportion index, risk prediction

## Abstract

**Objective:** To compare the circumferential proportion of mucosal defects, defect length, and the length-to-residual mucosal proportion (LRP) index for predicting esophageal stricture after endoscopic submucosal dissection (ESD) in patients with mucosal defects ≥6 cm, and to explore LRP in defects >10 cm. **Methods:** This retrospective study included 378 patients undergoing esophageal ESD from February 2014 to June 2020, grouped by stricture status. LRP was defined as defect length divided by residual mucosal proportion, with residual mucosal proportion set at a lower bound of 0.05 for statistical calculation. Receiver operating characteristic analysis compared the three indicators, including subgroup analysis for defects >10 cm. **Results:** Postoperative stricture occurred in 82 patients (21.7%). Circumferential proportion, defect length, and LRP were higher in the stricture group than in the non-stricture group (all *p* < 0.001). In the overall cohort, circumferential proportion and LRP showed comparable discrimination (AUC 0.877 vs. 0.876; *p* = 0.825), both outperforming defect length alone (AUC 0.694; both *p* < 0.001). In defects >10 cm, LRP showed the highest numerical AUC (0.913), followed by circumferential proportion (0.886) and defect length (0.668). Multivariable analysis confirmed that LRP was independently associated with stricture (per 10-unit increase: OR = 1.225; 95% CI, 1.150–1.304; *p* < 0.001). **Conclusions:** LRP was comparable to circumferential proportion and may serve as an exploratory complementary metric in long-segment defects, warranting external validation.

## 1. Introduction

Esophageal squamous cell carcinoma is a common gastrointestinal malignancy characterized by aggressive biological behavior, rapid progression, and poor overall prognosis, with the 5-year survival rate remaining unsatisfactory [1,2]. For patients with locally advanced or advanced esophageal squamous cell carcinoma, current treatments include surgery, chemoradiotherapy, and immunotherapy; however, long-term outcomes remain suboptimal, and the disease burden continues to be substantial [3,4]. By contrast, early esophageal squamous cell carcinoma, particularly lesions confined to the mucosa or superficial submucosa without definite evidence of lymph node metastasis, can achieve substantially improved outcomes after timely diagnosis and curative treatment, with recent studies reporting 5-year overall survival rates of 73–86% after treatment for superficial esophageal squamous cell carcinoma [5,6,7]. Therefore, early detection, diagnosis, and intervention are clinically important for improving long-term outcomes in patients with esophageal squamous cell carcinoma.

At present, commonly used treatment strategies for early esophageal squamous cell carcinoma include endoscopic and surgical resection. Among them, endoscopic resection has become an important standard therapeutic approach because it can preserve the anatomical structure and function of the esophagus [8,9]. Among available endoscopic techniques, esophageal endoscopic submucosal dissection (ESD) enables en bloc resection and accurate pathological assessment, while offering advantages such as minimal invasiveness, relatively few complications, and shorter hospital stay; consequently, it has been widely adopted in clinical practice [10,11].

In clinical practice, selection of endoscopic treatment for superficial esophageal squamous cell carcinoma should be based on a structured diagnostic work-up, including high-definition endoscopy with biopsy, image-enhanced endoscopy when appropriate, cross-sectional imaging to exclude nodal or distant disease, and endoscopic ultrasonography when invasion depth or submucosal extension is uncertain. Treatment decisions for lesions with suspected deep submucosal invasion, nodal disease, or other high-risk features should be considered within a multidisciplinary framework, in which endoscopic resection, surgery, neoadjuvant therapy, radiotherapy, systemic therapy, and surveillance can be individualized.

However, with the expansion of indications and resection extent, postoperative esophageal stricture has become one of the most clinically relevant adverse events after ESD, particularly after large, near-circumferential, or circumferential mucosal defects [12,13,14]. Once esophageal stricture develops, patients often present with dysphagia and may require repeated endoscopic dilatation, which markedly impairs quality of life and increases subsequent treatment burden [15,16,17]. Recent studies have shown that circumferential involvement remains a core predictor of stricture risk after esophageal ESD, while lesion length, longitudinal mucosal defect extent, and other lesion-related factors are also closely associated with stricture formation [17,18,19,20]. These findings suggest that esophageal stricture is not determined solely by transverse mucosal loss; in long-segment lesions, relying only on conventional circumferential assessment may not fully capture wound-healing burden and residual mucosal reserve [21,22]. Therefore, integrating longitudinal length information into traditional circumferential assessment may help identify patients at high risk of postoperative stricture and guide follow-up management more accurately.

Based on this rationale, the present study focused on risk assessment for esophageal stricture after ESD for superficial esophageal squamous cell carcinoma and compared the predictive performance of conventional circumferential proportion, mucosal defect length, and the LRP index. Particular attention was given to long-segment defects >10 cm, not to claim superiority of the LRP index, but to explore whether a metric integrating longitudinal defect burden and residual mucosal reserve could complement conventional circumferential assessment for individualized risk stratification.

## 2. Methods

### 2.1. Study Design and Population

This was a single-center retrospective study. Patients who underwent esophageal ESD in the Department of Gastroenterology, West China Hospital of Sichuan University, between February 2014 and June 2020 were reviewed. Eligible patients had pathologically confirmed early esophageal squamous cell carcinoma and complete perioperative and follow-up data. To focus on patients with relatively large mucosal defects and to minimize the influence of short lesions on the discriminatory performance of the evaluated indicators, only patients with mucosal defect length ≥6 cm were included in the final analysis. The inclusion criteria were as follows: (1) treatment with esophageal ESD; (2) postoperative pathological diagnosis of early esophageal cancer; (3) complete data on mucosal defect length, circumferential involvement, perioperative variables, and postoperative follow-up; and (4) follow-up data sufficient to determine whether postoperative esophageal stricture occurred. The exclusion criteria were as follows: (1) a history of esophageal surgery, radiotherapy, or other conditions that might affect the assessment of postoperative esophageal stricture; (2) concomitant diseases clearly affecting swallowing function or esophageal patency; (3) substantial missing perioperative data; and (4) loss to follow-up or insufficient follow-up to determine the outcome. This study was approved by the Ethics Committee of West China Hospital, Sichuan University (Approval No. 2024-shen-1465). As this was a retrospective chart-review study using only existing clinical data and involving no additional interventions, the requirement for written informed consent was waived by the ethics committee.

### 2.2. ESD Procedure and Perioperative Management

All ESD procedures were performed by experienced endoscopists at our center. Before ESD, patients routinely underwent endoscopy and relevant imaging examinations to assess lesion extent, invasion depth, and possible metastatic disease. During the procedure, lesion marking, submucosal injection, circumferential incision, and submucosal dissection were performed under direct endoscopic visualization until complete resection was achieved. During ESD, submucosal lifting was achieved using an injection solution composed of either normal saline, sodium hyaluronate, and methylene blue, or glycerol fructose, sodium hyaluronate, and methylene blue. After ESD, patients received standard postoperative management according to their clinical condition, including acid suppression, mucosal protection, and nutritional support. For patients with extensive postoperative mucosal defects or those judged clinically to be at high risk of stricture, prophylactic interventions such as postoperative oral steroids or intraoperative local steroid injection were selectively administered.

### 2.3. Data Collection

Clinical information, lesion characteristics, procedure-related variables, and outcome data were collected from the medical record system, endoscopy database, and follow-up records. Collected variables included: (1) general clinical data, such as age and sex; (2) lesion characteristics, including lesion location, mucosal defect length, circumferential involvement, postoperative pathological type, and invasion depth; (3) procedure-related variables, including en bloc resection, R0 resection, and prophylactic measures for stricture prevention; and (4) follow-up outcomes, including the occurrence of postoperative esophageal stricture, time to stricture, and treatment for postoperative stricture.

### 2.4. Definitions of Study Indicators

Mucosal defect length was defined as the longitudinal diameter of the post-ESD mucosal defect, measured in centimeters. Circumferential involvement was defined as the proportion of the esophageal circumference occupied by the postoperative mucosal defect and was analyzed as a continuous variable. Residual mucosal proportion was defined as the proportion of the esophageal circumference not involved by the mucosal defect and was calculated as 1 minus the circumferential involvement proportion. To integrate longitudinal wound burden and residual mucosal reserve, the length-to-residual mucosal proportion (LRP) index was defined as mucosal defect length (cm) divided by residual mucosal proportion. In the present study, the residual mucosal proportion was set at a lower bound of 0.05 for statistical calculation. This lower bound was used to avoid undefined or infinite values in complete circumferential defects, in which the observed residual mucosal proportion is zero, while retaining these clinically important high-risk cases in the analysis. A higher LRP index indicates greater longitudinal wound burden relative to the residual mucosal reserve and theoretically corresponds to a higher risk of postoperative esophageal stricture.

In addition, patients with mucosal defect length >10 cm were prespecified as a long-segment subgroup to further compare the discriminatory performance of conventional and newly constructed indicators in long lesions.

### 2.5. Outcome Definition and Follow-Up

The primary outcome was the occurrence of esophageal stricture after esophageal ESD. Esophageal stricture was defined as postoperative dysphagia or related clinical symptoms with endoscopically confirmed luminal narrowing, inability of a standard endoscope to pass smoothly through the stenotic segment, or the need for endoscopic dilatation. Follow-up was calculated from the date of ESD to the first occurrence of esophageal stricture, the last follow-up, or the end of the study follow-up period.

Follow-up information was obtained through outpatient visits, hospitalization records, and telephone follow-up.

### 2.6. Statistical Analysis

Statistical analyses were performed using SPSS version 27.0. Continuous variables are presented as mean ± standard deviation and were compared between groups using the independent-samples *t*-test, with Welch correction when equal variances were not assumed. Categorical variables are presented as numbers (percentages) and were compared using the chi-square test or Fisher’s exact test, as appropriate.

The main purpose of this study was to evaluate the predictive performance of conventional circumferential proportion, mucosal defect length, and the LRP index for esophageal stricture after ESD. First, differences in these indicators were compared between the stricture and non-stricture groups in the overall cohort. Receiver operating characteristic (ROC) curve analysis was then performed to evaluate the discriminatory ability of each indicator for postoperative esophageal stricture, and the area under the curve (AUC), 95% confidence interval (CI), optimal cutoff value, sensitivity, and specificity were calculated. Optimal cutoff values were determined using the Youden index, and differences between ROC curves were compared using the DeLong test.

To further assess the value of the LRP index in long-segment defects, subgroup analysis was performed in patients with mucosal defect length >10 cm, comparing mucosal defect length, circumferential proportion, and the LRP index. For multivariable logistic regression, the primary model included age, sex, lesion location, muscular injury, clip use, invasion depth, R0 resection status, and the LRP index. Procedure duration was available in 377 patients and was not included in the primary model in order to preserve the full cohort size; it was additionally evaluated in a sensitivity model using available cases. Stricture-prevention measures were not included in the primary regression model because prophylactic interventions were selectively administered according to clinical risk rather than a standardized protocol, and were therefore considered treatment-selection factors prone to confounding by indication; these measures were described and discussed as potential confounders. Odds ratios (ORs) and 95% CIs were calculated to determine whether the LRP index was independently associated with postoperative esophageal stricture. Sensitivity analyses were also performed using alternative lower bounds for residual mucosal proportion (0.01 and 0.10) and by excluding complete circumferential defects. All tests were two-sided, and *p* < 0.05 was considered statistically significant.

## 3. Results

### 3.1. Baseline Characteristics and Incidence of Postoperative Esophageal Stricture

A total of 378 patients who underwent esophageal ESD were included in the final analysis. Among them, 248 (65.6%) were male, and 130 (34.4%) were female, with a mean age of 62.29 ± 7.75 years. Lesions were most commonly located in the middle esophagus (n = 242, 64.0%), followed by the lower esophagus (n = 97, 25.7%) and upper esophagus (n = 39, 10.3%). Endoscopic submucosal tunnel dissection (ESTD) was the predominant procedure, performed in 368 patients (97.4%), and R0 resection was achieved in 359 patients (95.0%). According to the circumferential extent, <1/2, 1/2–<3/4, ≥3/4–<1, and circumferential defects were observed in 55 (14.6%), 171 (45.2%), 97 (25.7%), and 55 (14.6%) patients, respectively. The mean mucosal defect length was 8.06 ± 2.23 cm, and the mean LRP index was 51.05 ± 71.14.

Overall, 82 patients developed postoperative esophageal stricture, corresponding to an incidence of 21.7%. The incidence of postoperative stricture increased with circumferential involvement: 0.0% (0/55) in the <1/2 group, 8.2% (14/171) in the 1/2–<3/4 group, 26.8% (26/97) in the ≥3/4–<1 group, and 76.4% (42/55) in the circumferential group. In addition, all resected lesions were squamous carcinoma, and no adenocarcinoma was identified in the study cohort. Among the 378 patients, 288 (76.2%) had squamous cell carcinoma, 62 (16.4%) had high-grade dysplasia/high-grade intraepithelial neoplasia, and 28 (7.4%) had low-grade dysplasia/low-grade intraepithelial neoplasia.

### 3.2. Comparison Between the Stricture and Non-Stricture Groups

Compared with patients without postoperative esophageal stricture, those who developed stricture had longer procedure duration (110.30 ± 51.36 min vs. 71.54 ± 39.28 min, *p* < 0.001), higher mucosal defect circumferential proportion (0.88 ± 0.15 vs. 0.59 ± 0.18, *p* < 0.001), greater mucosal defect length (9.50 ± 3.13 cm vs. 7.66 ± 1.71 cm, *p* < 0.001), and a higher LRP index (130.15 ± 103.01 vs. 29.14 ± 36.52, *p* < 0.001). In addition, significant differences were observed between groups in sex, circumferential extent category, mucosal defect length >10 cm, lesion location, muscular injury, clip use, invasion depth, and R0 resection status, whereas age and procedure type did not differ significantly between groups (Table 1).

### 3.3. Predictive Performance of Each Indicator in the Overall Cohort

**Figure 1:**
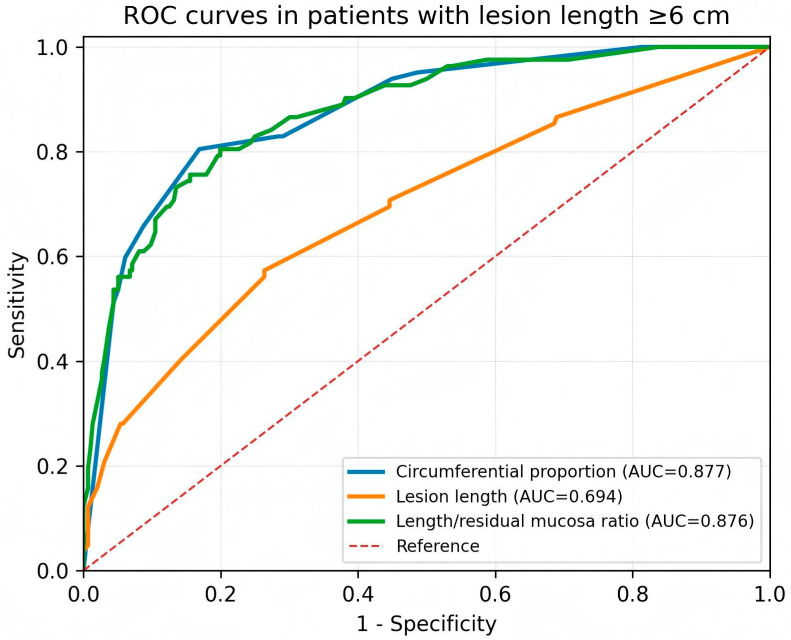
ROC curves for various predictors of postoperative esophageal stricture in the overall cohort.

ROC curve analysis in patients with mucosal defect length ≥6 cm showed that the mucosal defect circumferential proportion had the highest discriminatory ability for postoperative esophageal stricture, with an AUC of 0.877 (95% CI, 0.836–0.918). The optimal cutoff value was 0.80, with a sensitivity of 80.5% and specificity of 83.1%. The AUC of the LRP index was 0.876 (95% CI, 0.834–0.918), with an optimal cutoff value of 32.50, sensitivity of 80.5%, and specificity of 80.1%. Mucosal defect length alone showed a lower AUC of 0.694 (95% CI, 0.627–0.761), with an optimal cutoff value of 8.50 cm, sensitivity of 57.3%, and specificity of 73.6% (Figure 1). The DeLong test showed no significant difference in AUC between mucosal defect circumferential proportion and the LRP index (*p* = 0.825), whereas both indicators significantly outperformed mucosal defect length alone (both *p* < 0.001) (Table 2).

### 3.4. Predictive Performance in the Mucosal Defect Length >10 cm Subgroup

**Figure 2:**
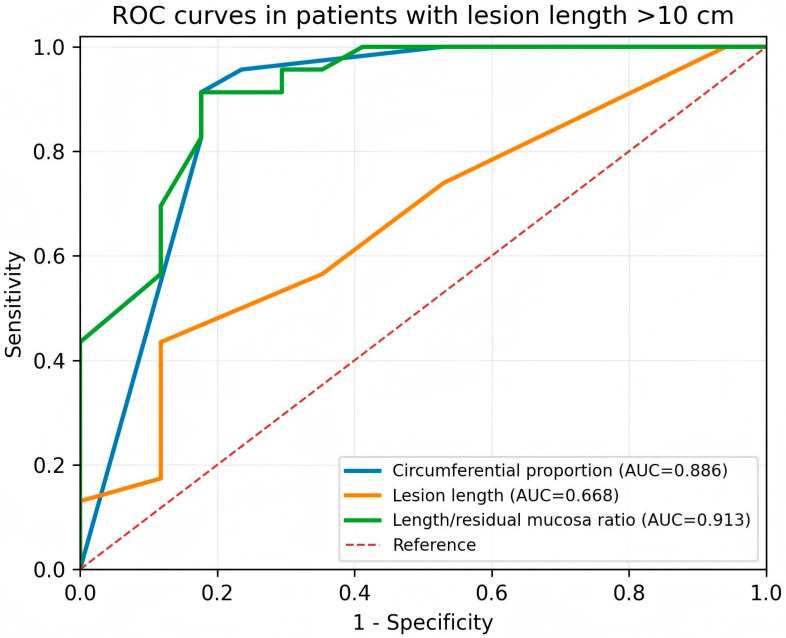
ROC curves for various predictors of postoperative esophageal stricture in the subgroup with mucosal defect length >10 cm.

In the prespecified long-segment subgroup with mucosal defect length >10 cm, 40 patients were included, of whom 23 developed postoperative esophageal stricture, corresponding to an incidence of 57.5%. In this subgroup, the LRP index had the highest AUC of 0.913 (95% CI, 0.823–1.000), with an optimal cutoff value of 110.00, sensitivity of 91.3%, and specificity of 82.4%. Mucosal defect circumferential proportion had an AUC of 0.886 (95% CI, 0.775–0.997), with an optimal cutoff value of 0.90, sensitivity of 91.3%, and specificity of 82.4%. Mucosal defect length alone had a relatively lower AUC of 0.668 (95% CI, 0.501–0.834) (Figure 2). The DeLong test showed that the LRP index was significantly superior to mucosal defect length alone (*p* = 0.002), and mucosal defect circumferential proportion was also superior to mucosal defect length alone (*p* = 0.023). Although the AUC of the LRP index was numerically higher than that of the mucosal defect circumferential proportion, the difference did not reach statistical significance (*p* = 0.290) (Table 3).

### 3.5. Multivariable Logistic Regression Analysis

Multivariable logistic regression analysis was performed to evaluate whether the LRP index was independently as-sociated with postoperative esophageal stricture. The primary model included age, sex, lesion location, muscular injury, clip use, invasion depth, R0 resection status, and the LRP index; the complete regression results are reported in Table 4. Procedure duration was available in 377 patients and was evaluated only in a sensitivity model using available cases, rather than in the primary model. Stricture-prevention measures were not included in the primary regression model because they were selectively administered according to clinical risk rather than by a standardized protocol, and were therefore prone to confounding by indication. Accordingly, they were described and discussed as potential treatment-selection confounders rather than presented as adjusted covariates in Table 4. In the primary model, the LRP index remained independently associated with postoperative esophageal stricture. For every 10-unit increase in the LRP index, the risk of postoperative esophageal stricture increased by approximately 22.5% (OR = 1.225; 95% CI, 1.150–1.304; *p* < 0.001). In addition, compared with upper esophageal lesions, lesions located in the middle esophagus (OR = 0.242; 95% CI, 0.096–0.607; *p* = 0.003) and lower esophagus (OR = 0.140; 95% CI, 0.042–0.473; *p* = 0.002) were associated with lower stricture risk. R0 resection was also associated with a lower risk of postopera-tive esophageal stricture (OR = 0.167; 95% CI, 0.042–0.662; *p* = 0.011). Sensitivity analyses using alternative lower bounds for residual mucosal proportion (0.01 and 0.10), as well as analyses excluding complete circumferential de-fects, yielded broadly consistent results (Supplementary Table S1).

## 4. Discussion

We agree that accurate pretreatment staging is fundamental in the management of esophageal cancer. The present study should therefore be interpreted within the context of patients who had already been selected for ESD after diagnostic endoscopy, pathological assessment, and imaging-based staging. Endoscopic ultrasonography may further improve assessment of invasion depth and submucosal spread in selected patients, particularly when biopsy findings alone are insufficient to define the extent of disease. These diagnostic steps are essential because they determine whether endoscopic treatment is appropriate or whether surgical, neoadjuvant, radiotherapeutic, or systemic strategies should be considered through multidisciplinary discussion.

In this study of 378 patients who underwent esophageal ESD with mucosal defect length ≥6 cm, we compared the predictive value of mucosal defect circumferential proportion, mucosal defect length, and the LRP index for postoperative esophageal stricture. The incidence of postoperative esophageal stricture was 21.7%, which is broadly consistent with previous large-scale studies of patients undergoing esophageal ESD [13,23]. In the overall cohort, the circumferential proportion of mucosal defects showed the highest discriminatory performance, with an AUC of 0.877, whereas the LRP index showed a nearly identical AUC of 0.876 and clearly outperformed mucosal defect length alone. These findings confirm that conventional circumferential assessment remains a robust core predictor in the overall population, while mucosal defect length alone has limited discriminatory value when used as an isolated predictor. Therefore, the LRP index should be interpreted as a complementary metric rather than a replacement for circumferential proportion.

Previous studies have repeatedly identified extensive circumferential mucosal defects as one of the most stable and important risk factors for esophageal stricture after ESD. Although most evidence on post-ESD esophageal stricture has historically come from East Asian centers, Western data are increasingly available and should be considered when interpreting the generalizability of risk stratification models. In a recent international Western multicenter study of circumferential ESD for early esophageal squamous cell carcinoma, de Santiago et al. reported that circumferential ESD was feasible in selected patients but was associated with a high risk of clinically relevant stricture despite frequent prophylactic measures, underscoring the need for better patient selection and more effective stricture-prevention strategies [24]. These Western data are directionally consistent with our finding that extensive circumferential and longitudinal mucosal defects represent a high-risk setting after esophageal ESD.

A recent meta-analysis also showed that circumferential resection extent, longitudinal lesion length, invasion depth, and other perioperative factors may increase stricture risk after esophageal ESD [13]. Consistent with these findings, our study showed a stepwise increase in postoperative stricture incidence with greater circumferential involvement, with the highest incidence observed in patients with circumferential defects. Thus, the newly proposed LRP index should not be interpreted as a replacement for conventional circumferential proportion; rather, it serves as a complementary metric that incorporates longitudinal wound burden into circumferential assessment.

Mechanistically, circumferential proportion primarily captures the transverse extent of mucosal loss, whereas stricture formation is also influenced by longitudinal wound extent, the total area of fibrotic contraction, and the burden of re-epithelialization [25]. For defects of short or moderate length, the width of the residual mucosal bridge may explain most stricture risk; however, in long-segment defects, similar circumferential proportions may correspond to substantially different wound areas and healing demands. The LRP index integrates mucosal defect length and residual mucosal reserve into a single expression, thereby reflecting both how long the defect extends and how much mucosa remains available for repair. This concept may better align with the clinical process of wound healing and scar contraction after long-segment esophageal ESD.

In the prespecified subgroup of patients with mucosal defect length >10 cm, the LRP index had an AUC of 0.913, numerically higher than that of the conventional circumferential proportion and substantially higher than that of mucosal defect length alone. However, the difference between the LRP index and circumferential proportion did not reach statistical significance; therefore, this subgroup finding should be interpreted as exploratory. Clinically, conventional circumferential proportion remains a simple and intuitive risk indicator, whereas the LRP index may provide additional descriptive information on longitudinal wound burden in selected long-segment defects. When the LRP index is markedly elevated, clinicians may consider closer surveillance and individualized prophylactic strategies, but such use should be validated in larger multicenter cohorts before being incorporated into routine decision-making. Recent studies have indicated that preventive approaches for post-ESD esophageal stricture include systemic or local steroids, stent placement, balloon dilatation, and regenerative medicine-based strategies; however, the benefit of these interventions depends on accurate risk stratification [12]. Steroid prophylaxis is particularly relevant because its selective use may confound the observed association between mucosal defect characteristics and subsequent stricture. In a Western center, Carpentier et al. reported a protocol using local triamcinolone injection for mucosal defects involving 50–89% of the circumference and add-on oral corticosteroids for resections involving ≥90% of the circumference, highlighting the clinical importance of steroid-based prevention after extensive esophageal ESD [26]. In the present cohort, steroid-based prophylaxis was not uniformly administered but was selectively adopted in clinically high-risk patients.

The present study focused on risk prediction before stricture formation and did not directly evaluate treatment strategies for established refractory strictures. In most post-ESD strictures, repeated endoscopic dilatation, steroid-based therapy, and other endoscopic approaches remain the main therapeutic options. However, for complex or difficult-to-resolve stenosis, minimally invasive surgery may be considered in highly selected cases. Recent literature has highlighted the role of robotic-assisted esophageal surgery in improving surgical precision and perioperative management in esophageal cancer treatment, although its role in post-ESD stricture management requires individualized assessment and should not be generalized to routine cases [27].

Multivariable analysis further showed that the LRP index remained independently associated with postoperative esophageal stricture, with each 10-unit increase corresponding to an approximately 22.5% increase in stricture risk. Lesion location and R0 resection status were also associated with postoperative stricture, consistent with previous prediction models that incorporated lesion site, invasion depth, and resection-related factors into risk assessment [23,28]. In the present study, upper esophageal lesions carried a higher risk, possibly because the upper esophagus has a relatively narrower lumen and limited anatomical space, making clinically relevant stenosis more likely under the same degree of scar contraction. Muscular injury was associated with stricture in univariable analysis but did not remain independently significant in the multivariable model. Because previous studies have suggested that muscular injury can increase post-ESD stricture risk, our finding should not be interpreted as evidence that muscular injury is clinically irrelevant; rather, its effect may have been confounded by defect extent, lesion complexity, and other adjusted variables [29].

In summary, the circumferential proportion of mucosal defects remains a core indicator for assessing esophageal stricture risk after ESD. The LRP index demonstrated comparable discriminatory ability in the overall cohort and a higher numerical AUC in long-segment defects >10 cm, but it did not show statistically significant superiority over circumferential proportion. These findings suggest that the LRP index may serve as a complementary exploratory metric for describing longitudinal wound burden and residual mucosal reserve in extensive defects, rather than as a replacement for conventional circumferential assessment.

## 5. Conclusions

In patients with a mucosal defect length of ≥6 cm after esophageal ESD, the mucosal defect circumferential proportion remains an important indicator for postoperative esophageal stricture risk, while the LRP index provides comparable discriminatory performance and clearly outperforms mucosal defect length alone. In long-segment defects >10 cm, the LRP index showed a higher numerical predictive performance, but the difference from the circumferential proportion was not statistically significant. Therefore, the LRP index should be viewed as a complementary and exploratory metric that integrates longitudinal wound burden and residual mucosal reserve, rather than as a superior substitute for conventional circumferential assessment. Future multicenter studies with larger sample sizes are warranted to validate the value of this index for postoperative stricture risk stratification and individualized prophylactic management.

## Figures and Tables

**Table 1:** Baseline and lesion characteristics of patients with mucosal defect length ≥6 cm according to the occurrence of esophageal stricture after esophageal ESD.

Variable	Overall (n = 378)	No Esophageal Stricture (n = 296)	Esophageal Stricture (n = 82)	*p* Value
Age, years	62.29 ± 7.75	62.05 ± 7.92	63.18 ± 7.09	0.213
Sex				0.005
Male	248 (65.6)	205 (69.3)	43 (52.4)	
Female	130 (34.4)	91 (30.7)	39 (47.6)	
Procedure type				1.000
ESD	10 (2.6)	8 (2.7)	2 (2.4)	
ESTD	368 (97.4)	288 (97.3)	80 (97.6)	
Procedure duration, min	79.95 ± 45.05	71.54 ± 39.28	110.30 ± 51.36	<0.001
Mucosal defect circumferential proportion	0.65 ± 0.21	0.59 ± 0.18	0.88 ± 0.15	<0.001
Circumferential extent category				<0.001
<1/2	55 (14.6)	55 (18.6)	0 (0.0)	
1/2–<3/4	171 (45.2)	157 (53.0)	14 (17.1)	
≥3/4–<1	97 (25.7)	71 (24.0)	26 (31.7)	
Circumferential	55 (14.6)	13 (4.4)	42 (51.2)	
Mucosal defect length, cm	8.06 ± 2.23	7.66 ± 1.71	9.50 ± 3.13	<0.001
Mucosal defect length >10 cm				<0.001
No	338 (89.4)	279 (94.3)	59 (72.0)	
Yes	40 (10.6)	17 (5.7)	23 (28.0)	
LRP index	51.05 ± 71.14	29.14 ± 36.52	130.15 ± 103.01	<0.001
Lesion location				<0.001
Upper esophagus	39 (10.3)	20 (6.8)	19 (23.2)	
Middle esophagus	242 (64.0)	189 (63.9)	53 (64.6)	
Lower esophagus	97 (25.7)	87 (29.4)	10 (12.2)	
Muscular injury				0.029
No	263 (69.6)	214 (72.3)	49 (59.8)	
Yes	115 (30.4)	82 (27.7)	33 (40.2)	
Clip use				0.016
No	355 (93.9)	283 (95.6)	72 (87.8)	
Yes	23 (6.1)	13 (4.4)	10 (12.2)	
Invasion depth				0.025
M1	185 (48.9)	155 (52.4)	30 (36.6)	
M2	78 (20.6)	59 (19.9)	19 (23.2)	
M3	65 (17.2)	42 (14.2)	23 (28.0)	
SM	47 (12.4)	38 (12.8)	9 (11.0)	
Not assessable/other	3 (0.8)	2 (0.7)	1 (1.2)	
R0 resection				<0.001
Yes	359 (95.0)	289 (97.6)	70 (85.4)	
No	19 (5.0)	7 (2.4)	12 (14.6)	

Note: Continuous variables are presented as mean ± standard deviation; categorical variables are presented as number (percentage). *p* values indicate comparisons between the no-stricture and stricture groups. Continuous variables were compared using Welch-corrected independent-samples *t*-tests, and categorical variables were compared using the chi-square test or Fisher’s exact test. LRP index = mucosal defect length/residual mucosal proportion (The lower bound of residual mucosal proportion was set at 0.05 in this study). ESD, endoscopic submucosal dissection; ESTD, endoscopic submucosal tunnel dissection; LRP, length-to-residual mucosal proportion; SM, submucosa.

**Table 2:** ROC analysis of different indicators for predicting postoperative esophageal stricture in patients with mucosal defect length ≥6 cm.

Indicator	AUC (95% CI)	Optimal Cutoff	Sensitivity	Specificity	*p* vs. Mucosal Defect Length	*p* vs. Circumferential Proportion
Mucosal defect circumferential proportion	0.877 (0.836–0.918)	0.80	80.5%	83.1%	<0.001	—
Mucosal defect length	0.694 (0.627–0.761)	8.50	57.3%	73.6%	—	<0.001
LRP index	0.876 (0.834–0.918)	32.50	80.5%	80.1%	<0.001	0.825

Note: AUCs and 95% CIs were calculated using the DeLong method. Optimal cutoff values were determined using the Youden index. AUC, area under the receiver operating characteristic curve; CI, confidence interval; LRP, length-to-residual mucosal proportion; ROC, receiver operating characteristic.

**Table 3:** ROC analysis of different indicators for predicting postoperative esophageal stricture in the mucosal defect length >10 cm subgroup.

Indicator	AUC (95% CI)	Optimal Cutoff	Sensitivity	Specificity	*p* vs. Mucosal Defect Length	*p* vs. Circumferential Proportion
Mucosal defect circumferential proportion	0.886 (0.775–0.997)	0.90	91.3%	82.4%	0.023	—
Mucosal defect length	0.668 (0.501–0.834)	13.75	43.5%	88.2%	—	0.023
LRP index	0.913 (0.823–1.000)	110.00	91.3%	82.4%	0.002	0.290

Note: AUCs and 95% CIs were calculated using the DeLong method. Optimal cutoff values were determined using the Youden index. AUC, area under the receiver operating characteristic curve; CI, confidence interval; LRP, length-to-residual mucosal proportion; ROC, receiver operating characteristic.

**Table 4:** Multivariable logistic regression analysis of factors associated with postoperative esophageal stricture.

Variable	Adjusted OR	95% CI	*p* Value
Age, per year	0.990	0.950–1.032	0.625
Male sex	0.840	0.405–1.744	0.640
LRP index, per 10-unit increase	1.225	1.150–1.304	<0.001
Middle esophagus vs. upper	0.242	0.096–0.607	0.002
Lower esophagus vs. upper	0.140	0.042–0.473	0.001
Muscular injury	1.077	0.523–2.220	0.840
Clip use	1.251	0.635–2.467	0.517
M2 vs. M1	1.367	0.586–3.187	0.469
M3 vs. M1	1.434	0.573–3.585	0.441
SM vs. M1	0.278	0.074–1.037	0.057
Other/unevaluable vs. M1	0.083	0.001–7.500	0.279
R0 resection	0.167	0.042–0.662	0.004

Note: OR, odds ratio; CI, confidence interval; LRP, length-to-residual mucosal proportion. The overall cohort included 378 patients with mucosal defect length ≥6 cm. Reference categories were female sex, upper esophagus, absence of muscular injury, absence of clip use, M1 invasion depth, and non-R0 resection. The LRP index was analyzed per 10-unit increase. The primary multivariable model included age, sex, lesion location, muscular injury, clip use, invasion depth, R0 resection status, and the LRP index. Procedure duration was available for 377 patients and was analyzed descriptively using available cases; to preserve the full cohort size, it was not included in the primary model, but a sensitivity model that additionally adjusted for procedure duration showed materially consistent results. Stricture-prevention measures were not included in the primary model because they were selectively administered according to clinical risk rather than by a standardized protocol and were therefore considered treatment-selection factors prone to confounding by indication.

## Data Availability

The Excel data supporting the findings of this study were provided by Kai Deng under a licensing agreement. To obtain this data, please contact Kai Deng at dengkai@wchscu.cn.

## Supplementary Materials

The following supporting information can be downloaded at: https://www.mdpi.com/article/10.3390/jcm15145445/s1, Table S1: Sensitivity and incremental analyses for the LRP index.

## References

[1] Yang H., Wang F., Hallemeier C.L., Lerut T., Fu J. (2024). Oesophageal cancer. Lancet.

[2] Jiang W., Zhang B., Xu J., Xue L., Wang L. (2025). Current status and perspectives of esophageal cancer: A comprehensive review. Cancer Commun..

[3] Qu H.T., Li Q., Hao L., Ni Y.J., Luan W.Y., Yang Z., Chen X.D., Zhang T.T., Miao Y.D., Zhang F. (2024). Esophageal cancer screening, early detection and treatment: Current insights and future directions. World J. Gastrointest. Oncol..

[4] Waters J.K., Reznik S.I. (2022). Update on Management of Squamous Cell Esophageal Cancer. Curr. Oncol. Rep..

[5] Sakanaka K. (2024). Treatment strategy for early-stage esophageal cancer. Jpn. J. Radiol..

[6] Wang X., Hobbs B., Gandhi S.J., Muijs C.T., Langendijk J.A., Lin S.H. (2021). Current status and application of proton therapy for esophageal cancer. Radiother. Oncol..

[7] Iizuka T. (2024). Curative criteria for endoscopic treatment of oesophageal squamous cell cancer. Best Pract. Res. Clin. Gastroenterol..

[8] di Pietro M., Canto M.I., Fitzgerald R.C. (2018). Endoscopic Management of Early Adenocarcinoma and Squamous Cell Carcinoma of the Esophagus: Screening, Diagnosis, and Therapy. Gastroenterology.

[9] Deboever N., Jones C.M., Yamashita K., Ajani J.A., Hofstetter W.L. (2024). Advances in diagnosis and management of cancer of the esophagus. BMJ.

[10] Zhang T., Wang H., Jin T., Wu Z., Li X., Zhang Q. (2024). Endoscopic submucosal dissection versus surgery for T1b esophageal carcinoma: A single-center retrospective study. J. Cancer Res. Clin. Oncol..

[11] Ke R.T., Hsiao Y.H., Tai W.C., Li S.H., Yao C.C., Chuang K.H., Lai H.H., Chen Y., Chen L.C., Lu H.I. (2024). Similar survival after endoscopic submucosal dissection and esophagectomy in early esophageal cancer and synchronous or metachronous head and neck cancer. J. Cardiothorac. Surg..

[12] Ye S., Hu J., Zhang D., Zhao S., Shi X., Li W., Wang J., Guan W., Yan L. (2024). Strategies for Preventing Esophageal Stenosis After Endoscopic Submucosal Dissection and Progress in Stem Cell-Based Therapies. Tissue Eng. Part B Rev..

[13] Lin N., Lin J., Gong J. (2021). Risk factors of postoperative stricture after endoscopic submucosal dissection for superficial esophageal neoplasms: A meta-analysis. Medicine.

[14] Zhou X., Wu B., Tang D., He Y., Fang L. (2025). Risk Prediction Models of Esophageal Strictures After Endoscopic Submucosal Dissection: A Systematic Review and Meta-Analysis. Dig. Dis. Sci..

[15] Uno K., Iijima K., Koike T., Shimosegawa T. (2015). Useful strategies to prevent severe stricture after endoscopic submucosal dissection for superficial esophageal neoplasm. World J. Gastroenterol..

[16] Isomoto H., Yamaguchi N., Nakayama T., Hayashi T., Nishiyama H., Ohnita K., Takeshima F., Shikuwa S., Kohno S., Nakao K. (2011). Management of esophageal stricture after complete circular endoscopic submucosal dissection for superficial esophageal squamous cell carcinoma. BMC Gastroenterol..

[17] Zou J., Chai N., Linghu E., Wang Z., Li L. (2022). Prevention of Esophageal Stricture After Whole Circumferential Endoscopic Resection: A Review for Endoscopists. Turk. J. Gastroenterol..

[18] Mizuno J., Urabe Y., Oka S., Konishi H., Ishibashi K., Fukuhara M., Tanaka H., Tsuboi A., Yamashita K., Hiyama Y. (2024). Predictive factors for esophageal stenosis in patients receiving prophylactic steroid therapy after endoscopic submucosal dissection for esophageal squamous cell carcinoma. BMC Gastroenterol..

[19] Dai N., Lu G.-F., Yin Y., Zhou Y.-Z., Zhao Y.-H., Ma J.-X., Cao X.-G., Guo C.-Q., Li Y.-R., Ren M.-D. (2025). Predictors of post-endoscopic submucosal dissection stricture in superficial esophageal cancer patients receiving oral steroid prophylaxis. Sci. Rep..

[20] Takahashi K., Fujiya M., Ueno N., Saito T., Sugiyama Y., Murakami Y., Iwama T., Sasaki T., Ijiri M., Tanaka K. (2019). White coat status is a predictive marker for post-esophageal endoscopic submucosal dissection stricture: A retrospective study. Esophagus.

[21] Mizuta H., Nishimori I., Kuratani Y., Higashidani Y., Kohsaki T., Onishi S. (2009). Predictive factors for esophageal stenosis after endoscopic submucosal dissection for superficial esophageal cancer. Dis. Esophagus.

[22] Chen M., Dang Y., Ding C., Yang J., Si X., Zhang G. (2020). Lesion size and circumferential range identified as independent risk factors for esophageal stricture after endoscopic submucosal dissection. Surg. Endosc..

[23] Xia S.Y., Lu Q., Wang Z.J., Gan T., Yang J.L., Wang Z. (2023). Development and validation of a model to determine the risk of esophageal strictures after endoscopic submucosal dissection for esophageal neoplasms. Surg. Endosc..

[24] Rodríguez de Santiago E., van Tilburg L., Deprez P.H., Pioche M., Pouw R.E., Bourke M.J., Seewald S., Weusten B.L.A.M., Jacques J., Leblanc S. (2024). Western outcomes of circumferential endoscopic submucosal dissection for early esophageal squamous cell carcinoma. Gastrointest. Endosc..

[25] Yang F., Hu Y., Shi Z., Liu M., Hu K., Ye G., Pang Q., Hou R., Tang K., Zhu Y. (2024). The occurrence and development mechanisms of esophageal stricture: State of the art review. J. Transl. Med..

[26] Carpentier D., Englebert G., Sanchez L.O., Bucalau A.-M., Verset L., Demetter P., Eisendrath P., Devière J., Lemmers A. (2024). Local triamcinolone injection and selective add-on oral steroids to prevent esophageal post-endoscopic submucosal dissection stricture: A retrospective analysis in a Western center. Endoscopy.

[27] Vashist Y., Goyal A., Shetty P., Girnyi S., Cwalinski T., Skokowski J., Malerba S., Prete F.P., Mocarski P., Kania M.K. (2025). Evaluating postoperative morbidity and outcomes of robotic-assisted esophagectomy in esophageal cancer treatment-a comprehensive review on behalf of TROGSS (the robotic global surgical society) and EFISDS (European federation international society for digestive surgery) joint working group. Curr. Oncol..

[28] Zhang M., Ma J., Tian W., Zhao N., Feng X., Lu P., Ding Q., Liu M. (2024). Prediction of post-ESD esophageal stricture by a nomogram and risk factor analysis of ineffective oral steroids prophylaxis. BMC Gastroenterol..

[29] Geng Z.H., Zhu Y., Li Q.L., Fu P.Y., Xiang A.Y., Pan H.T., Xu M.D., Chen S.Y., Zhong Y.S., Zhang Y.Q. (2023). Muscular injury as an independent risk factor for esophageal stenosis after endoscopic submucosal dissection of esophageal squamous cell cancer. Gastrointest. Endosc..

