# Supplementary material for: An Integrated Circumferential Proportion and Defect Length Index for Predicting Esophageal Stricture After ESD in Large Mucosal Defects ≥6 cm
Source: J Clin Med. 2026 Jul 11;15(14):5445. doi: 10.3390/jcm15145445 (PMC13412619; doi:10.3390/jcm15145445)
Supplement: Supplementary file 1 [file jcm-15-05445-s001.zip › jcm-4321278-supplementary.pdf]

Supplementary Table S1. Sensitivity and incremental analyses for the LRP index.

Analysis	Overall cohort AUC	>10 cm subgroup AUC	Interpretation
Primary calculation of LRP index: mucosal defect length divided by residual mucosal proportion, with the lower bound of residual mucosal proportion set at 0.05	0.876	0.913	Primary analysis
Alternative lower bound of residual mucosal proportion: 0.01	0.876	0.913	Comparable to the primary analysis
Alternative lower bound of residual mucosal proportion: 0.10	0.873	0.916	Comparable to the primary analysis
Excluding complete circumferential defects	0.796	0.902	Consistent but based on a different lower-risk population

Note. AUC, area under the receiver operating characteristic curve; LRP, length-to-residual mucosal proportion. The LRP index was defined as mucosal defect length divided by residual mucosal proportion. In the present study, the residual mucosal proportion was set at a lower bound of 0.05 for statistical calculation to avoid undefined or infinite values in complete circumferential defects while retaining these clinically important high-risk cases. The 0.01 and 0.10 analyses used alternative lower bounds of residual mucosal proportion.
